# Crystal structure of 1-[(*Z*)-2-phenyl­hydrazin-1-yl­idene]naphthalen-2(1*H*)-one

**DOI:** 10.1107/S2056989015006775

**Published:** 2015-04-09

**Authors:** Ali Benosmane, Mohamed Amine Benaouida, Assia Mili, Abdelkader Bouchoul, Hocine Merazig

**Affiliations:** a Unité de recherche de Chimie de l’Environnement et Moléculaire Structurale, Faculté du Sciences Exactes, Université de Constantine 1, 25000 Constantine, Algeria

**Keywords:** crystal structure, hydrazone, naphthalenone, hydrogen bonding, C—H⋯π inter­actions

## Abstract

In the title compound, C_16_H_12_N_2_O, the dihedral angle between the planes of the benzene ring and naphthalenone ring system is 1.89 (8)°; an intra­molecular N—H⋯O hydrogen bond occurs between the imino group and the carbonyl group. In the crystal, mol­ecules are linked by weak C—H⋯π inter­actions into supra­molecular chains propagating along the [01-1] direction.

## Related literature   

For general background to azo compounds and their use in dyes, pigments and advanced materials, see: Lee *et al.* (2004 ▸); Oueslati *et al.* (2004 ▸).
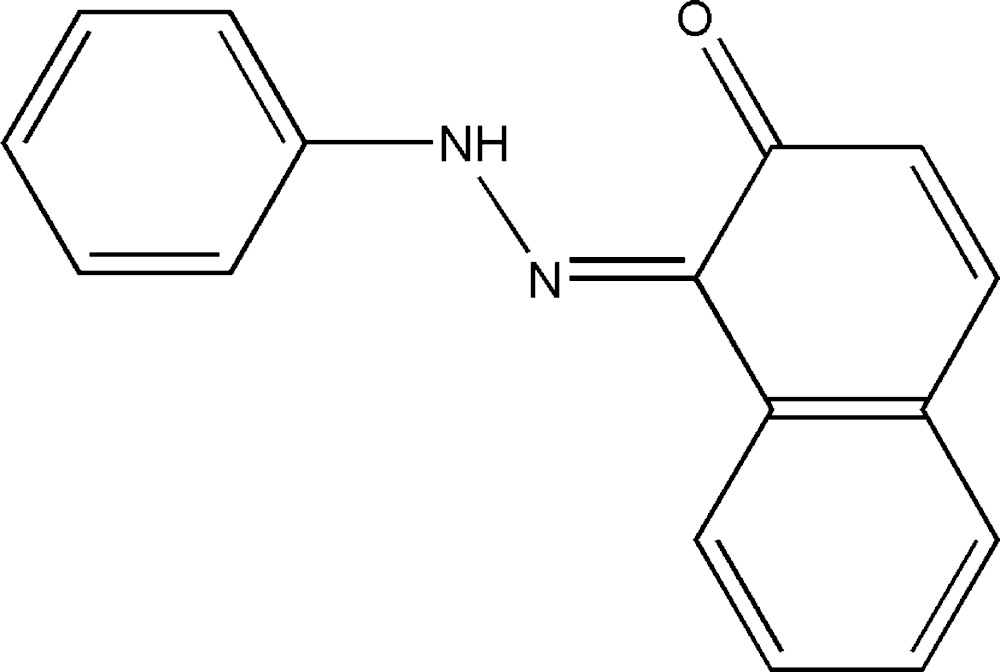


## Experimental   

### Crystal data  

C_16_H_12_N_2_O
*M* *_r_* = 248.28
Monoclinic, 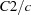

*a* = 28.109 (5) Å
*b* = 6.039 (5) Å
*c* = 15.181 (5) Å
β = 103.243 (5)°
*V* = 2508 (2) Å^3^
*Z* = 8
Mo *K*α radiation
μ = 0.08 mm^−1^
*T* = 293 K
0.09 × 0.04 × 0.01 mm

### Data collection  

Bruker APEXII diffractometer
4481 measured reflections
2450 independent reflections
1469 reflections with *I* > 2σ(*I*)
*R*_int_ = 0.034

### Refinement  

*R*[*F* ^2^ > 2σ(*F* ^2^)] = 0.057
*wR*(*F* ^2^) = 0.169
*S* = 1.05
2450 reflections
176 parameters
2 restraints
H atoms treated by a mixture of independent and constrained refinement
Δρ_max_ = 0.20 e Å^−3^
Δρ_min_ = −0.20 e Å^−3^

### 

Data collection: *APEX2* (Bruker, 2006 ▸); cell refinement: *SAINT* (Bruker, 2006 ▸); data reduction: *SAINT*; program(s) used to solve structure: *SHELXS97* (Sheldrick, 2008 ▸); program(s) used to refine structure: *SHELXL97* (Sheldrick, 2008 ▸); molecular graphics: *ORTEP-3 for Windows* (Farrugia, 2012 ▸) and *PLATON* (Spek, 2009 ▸); software used to prepare material for publication: *PLATON*.

## Supplementary Material

Crystal structure: contains datablock(s) global, I. DOI: 10.1107/S2056989015006775/xu5845sup1.cif

Structure factors: contains datablock(s) I. DOI: 10.1107/S2056989015006775/xu5845Isup2.hkl

Supporting information file. DOI: 10.1107/S2056989015006775/xu5845Isup3.cml

**Fig. 1.**
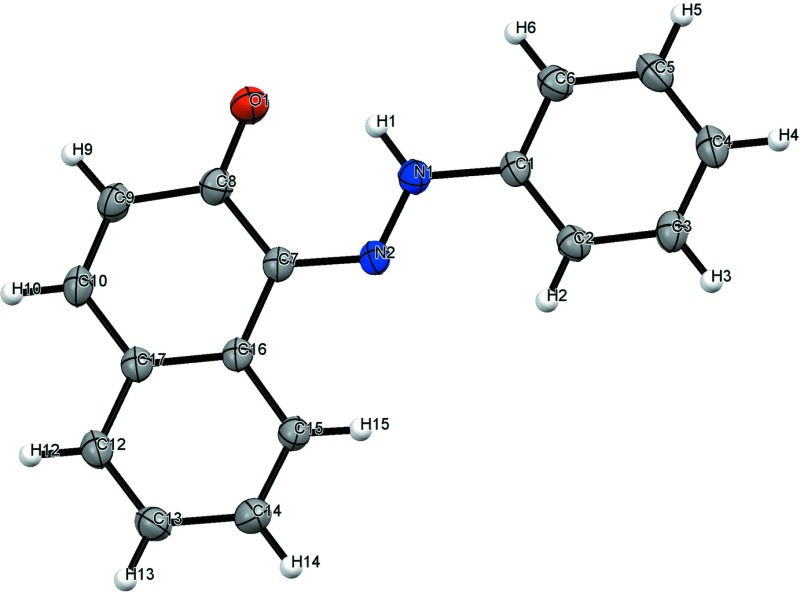
The molecular structure of the title molecule with the atom-numbering scheme. Ellipsoids are drawn at the 50% probability level and H atoms are shown as small spheres of arbitrary radii.

. DOI: 10.1107/S2056989015006775/xu5845fig1.tif
The mol­ecular structure of the title mol­ecule with the atom-numbering scheme. Ellipsoids are drawn at the 50% probability level and H atoms are shown as small spheres of arbitrary radii.

CCDC reference: 1057924

Additional supporting information:  crystallographic information; 3D view; checkCIF report

## Figures and Tables

**Table 1:** Hydrogen-bond geometry (, ) *Cg*1 and *Cg*3 are the centroids of the C1C6 and C12C17 rings, respectively.

*D*H*A*	*D*H	H*A*	*D* *A*	*D*H*A*
N1H1O1	0.906(17)	1.81(2)	2.550(3)	137(2)
C4H4*Cg*3^i^	0.93	2.76	3.568(4)	145
C12H12*Cg*1^ii^	0.93	2.83	3.612(4)	142

Symmetry codes: (i) 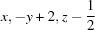; (ii) 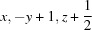.

## supplementary crystallographic information

### S1. Comment

The azo dyes are by far the most important class, accounting for over 50% of all commercial dyes, and having been studied more than other class (Lee *et al.*, 2004; Oueslati *et al.*, 2004). Azo dyes contain at least one azo group (–N=N–) but can contain two (diazo), three(triazo), or, more rarely, four (tetrakisazo) or more (polyazo) azo groups. The azo group is attached to two groups, of which at least one, but more usually both are aromatic. They exist in the Trans form in which the band angle is 120°, the nitrogen atoms are sp^2^ hybridized. Almost without exception, azo dyes are made by diazotization of primary aromatic amine followed by coupling of the resultant diazonium salt with an electron-richnucleophile. We report here in the crystal structure of the title compound, obtained through the diazotization of aniline followed by a coupling reaction with2-naphthol.

The molecular structure of (I) and the atom-numbering scheme are shown in Figure 1. Two aromatic rings A (C1—C6) and B (C7—C16) show a little deviation from planarity with a dihedral angle of 1.56°. Intramolecular hydrogen bonds are formed between the phenol hydroxyl groups and the nearest N atom in the aminobenze groups (Table 1).

### S2. Experimental

Treatment of aniline (0.02 mol) in 6 ml of 12*M* HCl and NaNO_2_ (0.0214 mol) in 8 ml of H_2_O for 30 min. To the obtained solution, was added dropwise a solution of naphthalen-2-ol, and the resulting brown precipitates were filtrated and washed with water, and dried in a desiccator for several days. Single crystals were obtained by slow evaporation from a pentane solution.

### S3. Refinement

The imino-H atom was located in a difference Fourier map and refined freely with *U*_iso_(H) = 1.2*U*_eq_(N). Other H atoms were placed in geometrically idealized positions and refined as riding, with C—H = 0.93 Å and *U*_iso_(H) = 1.2*U*_eq_(C).

### Crystal data

**Table:**

C_16_H_12_N_2_O	*Z* = 8
*M**_r_* = 248.28	*F*(000) = 1040
Monoclinic, *C*2/*c*	*D*_x_ = 1.315 Mg m^−^^3^
Hall symbol: -C 2yc	Mo *K*α radiation, λ = 0.71073 Å
*a* = 28.109 (5) Å	µ = 0.08 mm^−^^1^
*b* = 6.039 (5) Å	*T* = 293 K
*c* = 15.181 (5) Å	Needle, red
β = 103.243 (5)°	0.09 × 0.04 × 0.01 mm
*V* = 2508 (2) Å^3^	

### Data collection

**Table:**

Bruker APEXII diffractometer	*R*_int_ = 0.034
Horizonally mounted graphite crystal monochromator	θ_max_ = 26.0°, θ_min_ = 2.8°
CCD rotation images, thick slices scans	*h* = −33→34
4481 measured reflections	*k* = −7→7
2450 independent reflections	*l* = −18→18
1469 reflections with *I* > 2σ(*I*)	

### Refinement

**Table:**

Refinement on *F*^2^	Primary atom site location: structure-invariant direct methods
Least-squares matrix: full	Secondary atom site location: difference Fourier map
*R*[*F*^2^ > 2σ(*F*^2^)] = 0.057	Hydrogen site location: inferred from neighbouring sites
*wR*(*F*^2^) = 0.169	H atoms treated by a mixture of independent and constrained refinement
*S* = 1.05	*w* = 1/[σ^2^(*F*_o_^2^) + (0.092*P*)^2^] where *P* = (*F*_o_^2^ + 2*F*_c_^2^)/3
2450 reflections	(Δ/σ)_max_ < 0.001
176 parameters	Δρ_max_ = 0.20 e Å^−^^3^
2 restraints	Δρ_min_ = −0.20 e Å^−^^3^

### Special details

**Table:**

Geometry. Bond distances, angles *etc*. have been calculated using the rounded fractional coordinates. All su's are estimated from the variances of the (full) variance-covariance matrix. The cell e.s.d.'s are taken into account in the estimation of distances, angles and torsion angles
Refinement. Refinement of *F*^2^ against ALL reflections. The weighted *R*-factor *wR* and goodness of fit *S* are based on *F*^2^, conventional *R*-factors *R* are based on *F*, with *F* set to zero for negative *F*^2^. The threshold expression of *F*^2^ > σ(*F*^2^) is used only for calculating *R*-factors(gt) *etc*. and is not relevant to the choice of reflections for refinement. *R*-factors based on *F*^2^ are statistically about twice as large as those based on *F*, and *R*- factors based on ALL data will be even larger.

### Fractional atomic coordinates and isotropic or equivalent isotropic displacement parameters (Å^2^)

**Table:**

	*x*	*y*	*z*	*U*_iso_*/*U*_eq_	
O1	0.73024 (5)	0.6381 (3)	0.09280 (11)	0.0762 (6)	
N1	0.65766 (7)	0.8669 (3)	0.00890 (12)	0.0551 (6)	
N2	0.62773 (6)	0.7373 (2)	0.03831 (10)	0.0496 (5)	
C1	0.63841 (7)	1.0493 (3)	−0.04642 (12)	0.0507 (6)	
C2	0.58893 (8)	1.0917 (3)	−0.07177 (13)	0.0581 (7)	
C3	0.57264 (9)	1.2751 (3)	−0.12414 (14)	0.0647 (8)	
C4	0.60525 (10)	1.4165 (3)	−0.15058 (14)	0.0668 (9)	
C5	0.65432 (10)	1.3745 (3)	−0.12602 (15)	0.0708 (9)	
C6	0.67128 (8)	1.1886 (3)	−0.07340 (14)	0.0627 (8)	
C7	0.64663 (7)	0.5645 (3)	0.09114 (12)	0.0474 (6)	
C8	0.69840 (7)	0.5157 (3)	0.11735 (13)	0.0571 (7)	
C9	0.71329 (8)	0.3216 (3)	0.17167 (14)	0.0663 (8)	
C10	0.68014 (8)	0.1878 (3)	0.19510 (14)	0.0629 (8)	
C12	0.59514 (8)	0.0806 (3)	0.19309 (14)	0.0618 (8)	
C13	0.54622 (8)	0.1209 (3)	0.16897 (15)	0.0667 (8)	
C14	0.52907 (8)	0.3124 (3)	0.12094 (14)	0.0642 (8)	
C15	0.56100 (7)	0.4592 (3)	0.09687 (13)	0.0560 (7)	
C16	0.61175 (7)	0.4199 (3)	0.11928 (12)	0.0465 (6)	
C17	0.62863 (7)	0.2274 (3)	0.16940 (12)	0.0511 (6)	
H1	0.6900 (5)	0.835 (4)	0.022 (2)	0.127 (11)*	
H2	0.56680	0.99750	−0.05370	0.0700*	
H3	0.53930	1.30370	−0.14180	0.0780*	
H4	0.59390	1.54080	−0.18520	0.0800*	
H5	0.67630	1.46940	−0.14430	0.0850*	
H6	0.70460	1.15890	−0.05660	0.0750*	
H9	0.74640	0.28840	0.19080	0.0800*	
H10	0.69120	0.06310	0.22980	0.0750*	
H12	0.60650	−0.04640	0.22580	0.0740*	
H13	0.52430	0.02100	0.18450	0.0800*	
H14	0.49570	0.34080	0.10510	0.0770*	
H15	0.54900	0.58690	0.06520	0.0670*	

### Atomic displacement parameters (Å^2^)

**Table:**

	*U*^11^	*U*^22^	*U*^33^	*U*^12^	*U*^13^	*U*^23^
O1	0.0554 (9)	0.0765 (10)	0.0933 (12)	−0.0043 (7)	0.0103 (8)	0.0133 (8)
N1	0.0601 (11)	0.0475 (9)	0.0584 (11)	0.0026 (8)	0.0153 (9)	0.0031 (7)
N2	0.0601 (10)	0.0441 (8)	0.0455 (9)	0.0048 (7)	0.0139 (8)	−0.0013 (7)
C1	0.0666 (13)	0.0414 (9)	0.0457 (11)	0.0035 (9)	0.0160 (9)	−0.0021 (8)
C2	0.0695 (14)	0.0496 (11)	0.0568 (13)	0.0017 (9)	0.0180 (11)	0.0021 (9)
C3	0.0791 (15)	0.0538 (11)	0.0594 (13)	0.0129 (11)	0.0119 (12)	0.0008 (10)
C4	0.0995 (19)	0.0508 (11)	0.0515 (13)	0.0121 (12)	0.0201 (12)	0.0054 (9)
C5	0.1042 (19)	0.0529 (11)	0.0638 (14)	−0.0047 (12)	0.0371 (13)	0.0041 (10)
C6	0.0723 (14)	0.0594 (12)	0.0607 (13)	−0.0004 (10)	0.0244 (11)	0.0013 (10)
C7	0.0555 (11)	0.0441 (9)	0.0411 (10)	0.0051 (8)	0.0080 (9)	−0.0020 (8)
C8	0.0557 (13)	0.0568 (11)	0.0553 (12)	0.0009 (10)	0.0057 (10)	−0.0013 (9)
C9	0.0566 (13)	0.0693 (13)	0.0664 (14)	0.0093 (11)	0.0003 (11)	0.0102 (11)
C10	0.0722 (15)	0.0562 (12)	0.0566 (12)	0.0164 (10)	0.0069 (11)	0.0111 (10)
C12	0.0806 (16)	0.0512 (11)	0.0556 (12)	0.0085 (10)	0.0198 (11)	0.0076 (9)
C13	0.0737 (15)	0.0608 (12)	0.0719 (15)	−0.0033 (11)	0.0297 (12)	0.0048 (11)
C14	0.0583 (13)	0.0662 (13)	0.0704 (14)	0.0050 (10)	0.0195 (11)	0.0013 (11)
C15	0.0603 (13)	0.0524 (10)	0.0565 (12)	0.0098 (9)	0.0161 (10)	0.0056 (9)
C16	0.0568 (12)	0.0436 (9)	0.0394 (9)	0.0064 (8)	0.0117 (8)	−0.0039 (8)
C17	0.0627 (13)	0.0468 (10)	0.0438 (10)	0.0060 (9)	0.0123 (9)	−0.0011 (8)

### Geometric parameters (Å, º)

**Table:**

O1—C8	1.280 (3)	C12—C13	1.361 (3)
N1—N2	1.301 (3)	C13—C14	1.393 (3)
N1—C1	1.415 (3)	C14—C15	1.369 (3)
N2—C7	1.349 (3)	C15—C16	1.409 (3)
N1—H1	0.906 (17)	C16—C17	1.411 (3)
C1—C2	1.379 (3)	C2—H2	0.9300
C1—C6	1.379 (3)	C3—H3	0.9300
C2—C3	1.379 (3)	C4—H4	0.9300
C3—C4	1.378 (4)	C5—H5	0.9300
C4—C5	1.368 (4)	C6—H6	0.9300
C5—C6	1.397 (3)	C9—H9	0.9300
C7—C8	1.448 (3)	C10—H10	0.9300
C7—C16	1.449 (3)	C12—H12	0.9300
C8—C9	1.439 (3)	C13—H13	0.9300
C9—C10	1.342 (3)	C14—H14	0.9300
C10—C17	1.431 (3)	C15—H15	0.9300
C12—C17	1.399 (3)		
			
O1···N1	2.550 (3)	C4···H12^iv^	2.9200
O1···N2	2.873 (3)	C5···H12^iv^	3.0700
O1···H1	1.81 (2)	C8···H1	2.39 (3)
O1···H6^i^	2.7100	C12···H4^vi^	2.9400
N1···O1	2.550 (3)	C13···H4^vi^	3.0800
N1···C10^ii^	3.366 (4)	C14···H3^v^	3.0700
N2···O1	2.873 (3)	C17···H4^vi^	2.9600
N2···C4^iii^	3.398 (4)	H1···O1	1.81 (2)
N2···C12^ii^	3.412 (4)	H1···C8	2.39 (3)
N2···H2	2.5000	H1···H6	2.3800
N2···H15	2.5100	H2···N2	2.5000
C1···C16^ii^	3.572 (4)	H3···C14^v^	3.0700
C1···C17^ii^	3.519 (4)	H3···H14^v^	2.4800
C2···C16^ii^	3.450 (4)	H4···C12^vii^	2.9400
C4···N2^ii^	3.398 (4)	H4···C13^vii^	3.0800
C5···C7^ii^	3.544 (4)	H4···C17^vii^	2.9600
C6···C8^ii^	3.443 (4)	H6···H1	2.3800
C6···C7^ii^	3.559 (4)	H6···O1^i^	2.7100
C7···C5^iii^	3.544 (4)	H9···H10^viii^	2.5100
C7···C6^iii^	3.559 (4)	H10···H12	2.4600
C8···C6^iii^	3.443 (4)	H10···H9^ix^	2.5100
C10···N1^iii^	3.366 (4)	H12···H10	2.4600
C12···N2^iii^	3.412 (4)	H12···C3^x^	3.0000
C16···C1^iii^	3.572 (4)	H12···C4^x^	2.9200
C16···C2^iii^	3.450 (4)	H12···C5^x^	3.0700
C17···C1^iii^	3.519 (4)	H14···C3^v^	3.0700
C3···H12^iv^	3.0000	H14···H3^v^	2.4800
C3···H14^v^	3.0700	H15···N2	2.5100
			
N2—N1—C1	118.85 (18)	C7—C16—C15	122.93 (17)
N1—N2—C7	118.17 (17)	C12—C17—C16	119.86 (18)
N2—N1—H1	119.5 (16)	C10—C17—C12	121.51 (17)
C1—N1—H1	121.6 (16)	C10—C17—C16	118.63 (17)
N1—C1—C2	122.28 (18)	C1—C2—H2	120.00
N1—C1—C6	117.38 (18)	C3—C2—H2	120.00
C2—C1—C6	120.34 (18)	C2—C3—H3	120.00
C1—C2—C3	119.4 (2)	C4—C3—H3	120.00
C2—C3—C4	120.7 (2)	C3—C4—H4	120.00
C3—C4—C5	120.15 (19)	C5—C4—H4	120.00
C4—C5—C6	119.7 (2)	C4—C5—H5	120.00
C1—C6—C5	119.7 (2)	C6—C5—H5	120.00
C8—C7—C16	120.11 (16)	C1—C6—H6	120.00
N2—C7—C8	123.70 (17)	C5—C6—H6	120.00
N2—C7—C16	116.15 (17)	C8—C9—H9	120.00
C7—C8—C9	117.72 (18)	C10—C9—H9	120.00
O1—C8—C9	120.41 (19)	C9—C10—H10	118.00
O1—C8—C7	121.87 (17)	C17—C10—H10	118.00
C8—C9—C10	120.9 (2)	C13—C12—H12	120.00
C9—C10—C17	123.23 (18)	C17—C12—H12	119.00
C13—C12—C17	121.02 (18)	C12—C13—H13	120.00
C12—C13—C14	119.8 (2)	C14—C13—H13	120.00
C13—C14—C15	120.5 (2)	C13—C14—H14	120.00
C14—C15—C16	121.05 (18)	C15—C14—H14	120.00
C7—C16—C17	119.28 (18)	C14—C15—H15	119.00
C15—C16—C17	117.77 (17)	C16—C15—H15	119.00
			
C1—N1—N2—C7	−179.84 (16)	C8—C7—C16—C15	179.34 (17)
N2—N1—C1—C2	−1.4 (3)	C8—C7—C16—C17	−2.4 (3)
N2—N1—C1—C6	177.41 (17)	O1—C8—C9—C10	−177.61 (19)
N1—N2—C7—C8	0.3 (3)	C7—C8—C9—C10	1.7 (3)
N1—N2—C7—C16	−177.64 (16)	C8—C9—C10—C17	−0.6 (3)
N1—C1—C2—C3	178.57 (18)	C9—C10—C17—C12	178.22 (19)
C6—C1—C2—C3	−0.2 (3)	C9—C10—C17—C16	−2.0 (3)
N1—C1—C6—C5	−178.28 (18)	C17—C12—C13—C14	−0.8 (3)
C2—C1—C6—C5	0.5 (3)	C13—C12—C17—C10	179.41 (19)
C1—C2—C3—C4	−0.5 (3)	C13—C12—C17—C16	−0.4 (3)
C2—C3—C4—C5	0.9 (3)	C12—C13—C14—C15	0.8 (3)
C3—C4—C5—C6	−0.5 (3)	C13—C14—C15—C16	0.5 (3)
C4—C5—C6—C1	−0.2 (3)	C14—C15—C16—C7	176.67 (18)
N2—C7—C8—O1	1.2 (3)	C14—C15—C16—C17	−1.7 (3)
N2—C7—C8—C9	−178.06 (17)	C7—C16—C17—C10	3.4 (3)
C16—C7—C8—O1	179.10 (18)	C7—C16—C17—C12	−176.80 (17)
C16—C7—C8—C9	−0.2 (3)	C15—C16—C17—C10	−178.21 (17)
N2—C7—C16—C15	−2.6 (3)	C15—C16—C17—C12	1.6 (3)
N2—C7—C16—C17	175.67 (16)		

Symmetry codes: (i) −*x*+3/2, −*y*+3/2, −*z*; (ii) *x*, *y*+1, *z*; (iii) *x*, *y*−1, *z*; (iv) *x*, −*y*+1, *z*−1/2; (v) −*x*+1, −*y*+2, −*z*; (vi) *x*, −*y*+2, *z*+1/2; (vii) *x*, −*y*+2, *z*−1/2; (viii) −*x*+3/2, *y*+1/2, −*z*+1/2; (ix) −*x*+3/2, *y*−1/2, −*z*+1/2; (x) *x*, −*y*+1, *z*+1/2.

### Hydrogen-bond geometry (Å, º)

Cg1 and Cg3 are the centroids of the C1–C6 and C12–C17 rings, respectively.

**Table:**

*D*—H···*A*	*D*—H	H···*A*	*D*···*A*	*D*—H···*A*
N1—H1···O1	0.906 (17)	1.81 (2)	2.550 (3)	137 (2)
C4—H4···*Cg*3^vii^	0.93	2.76	3.568 (4)	145
C12—H12···*Cg*1^x^	0.93	2.83	3.612 (4)	142

Symmetry codes: (vii) *x*, −*y*+2, *z*−1/2; (x) *x*, −*y*+1, *z*+1/2.
