# Supplementary figures and images for: Crystal structure of 1-[(Z)-2-phenyl­hydrazin-1-yl­idene]naphthalen-2(1H)-one
Source: Acta Crystallogr E Crystallogr Commun. 2015 Apr 9;71(Pt 5):o303. doi: 10.1107/S2056989015006775 (PMC4420051; doi:10.1107/S2056989015006775)

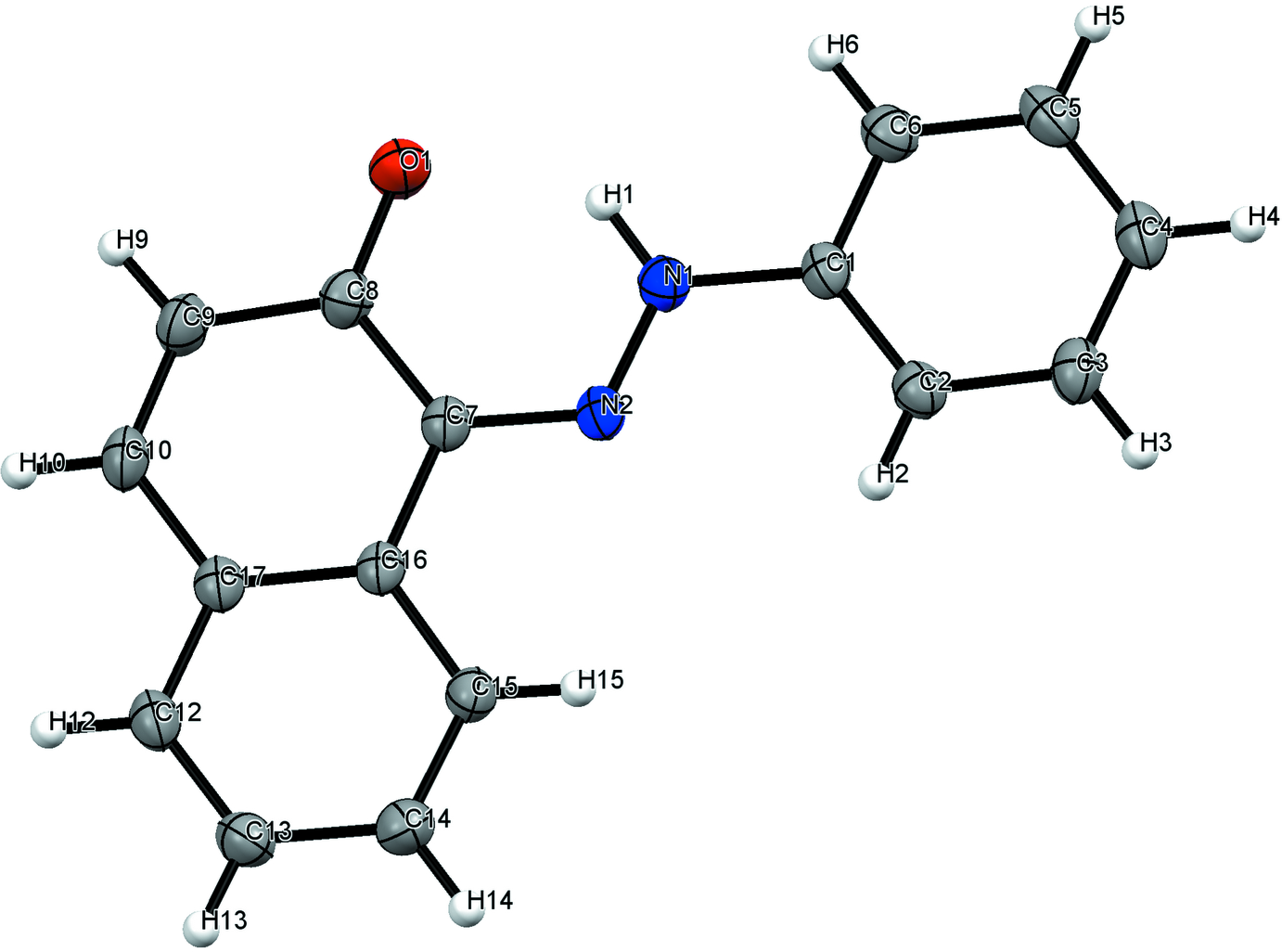

Supplement: Supplementary file 4 [file e-71-0o303-fig1.tif]
